# Poster Session II - A316 LONG-TERM OUTCOMES OF ENDOSCOPIC DRAINAGE MODALITIES IN PATIENTS WITH PANCREATIC AND PERIPANCREATIC FLUID COLLECTIONS FOLLOWING ACUTE PANCREATITIS: AN INTERNATIONAL MULTICENTER COHORT STUD

**DOI:** 10.1093/jcag/gwaf042.316

**Published:** 2026-02-13

**Authors:** K Khalaf, K Pawlak, M Jagielski, M Terrin, J Mosko, C W Teshima, G May, M Jackowski, A Repici, N Calo

**Affiliations:** Division of Gastroenterology, St. Michael’s Hospital, University of Toronto, Toronto, ON, Canada; Division of Gastroenterology, St. Michael’s Hospital, University of Toronto, Toronto, ON, Canada; Uniwersytet Mikolaja Kopernika w Toruniu, Torun, Kuyavian-Pomeranian Voivodeship, Poland; IRCCS Humanitas Research Hospital Cancer Centre, Rozzano, Lombardia, Italy; Division of Gastroenterology, St. Michael’s Hospital, University of Toronto, Toronto, ON, Canada; Division of Gastroenterology, St. Michael’s Hospital, University of Toronto, Toronto, ON, Canada; Division of Gastroenterology, St. Michael’s Hospital, University of Toronto, Toronto, ON, Canada; Uniwersytet Mikolaja Kopernika w Toruniu, Torun, Kuyavian-Pomeranian Voivodeship, Poland; IRCCS Humanitas Research Hospital Cancer Centre, Rozzano, Lombardia, Italy; Division of Gastroenterology, St. Michael’s Hospital, University of Toronto, Toronto, ON, Canada

## Abstract

**Background:**

The clinical trajectory of patients with peripancreatic collections is heterogenous and long-term outcomes following endoscopic drainage remain poorly characterized.

**Aims:**

This study aims to evaluate long-term outcomes in patients with PPFC who underwent different drainage modalities and determine independent predictors of time to clinical improvement.

**Methods:**

Consecutive adult (age ≥ 18 years) patients with history of acute pancreatitis and radiologically confirmed PPFC who underwent endoscopic drainage were included. PPFC were categorized as simple (localized collections) or complex (extending into the paracolic space). The primary outcome was time to clinical improvement, defined as resolution of infection, biliary or gastric outlet obstruction, or pain. Secondary outcomes included predictors of clinical improvement and overall PPFC resolution.

**Results:**

A total of 404 patients were included in this report. The mean age was 54.18 years (SD 15.3), 68.81% were male and 254 (62.9%) had complex collections. LAMS or BFMS placement was associated with a faster time to improvement (HR = 1.95, 95% CI: 1.32-2.90, p = 0.001), whereas complex collections (HR = 0.30, 95% CI: 0.21-0.44, p < 0.001) and prolonged hospitalization (HR = 0.99, 95% CI: 0.98-0.99, p < 0.001) were predictors of delayed clinical improvement. In comparison with patients with simple collections, those with complex collections had lower collection resolution rates (68.62% vs. 88.41%, p < 0.001) as well as higher rates of readmission (median of 3 [IQR: 0–5]) vs 3 [IQR: 0–3], p = 0.03), multiorgan failure (15.8% vs 1.3%, p < 0.001), and portal vein thrombosis (7.1% vs. 2.0%, p = 0.02).

**Conclusions:**

Patients with complex collections, extending into the paracolic space, had prolonged recovery and increased intervention requirements. In these patients, metal stent (LAMS or BFMS) placement significantly accelerated clinical improvement. Further research is needed to refine management strategies for these high-risk patients.

A316 Table 1: Results of Cox Proportional Hazards Regression Analysis for Time to Clinical Improvement

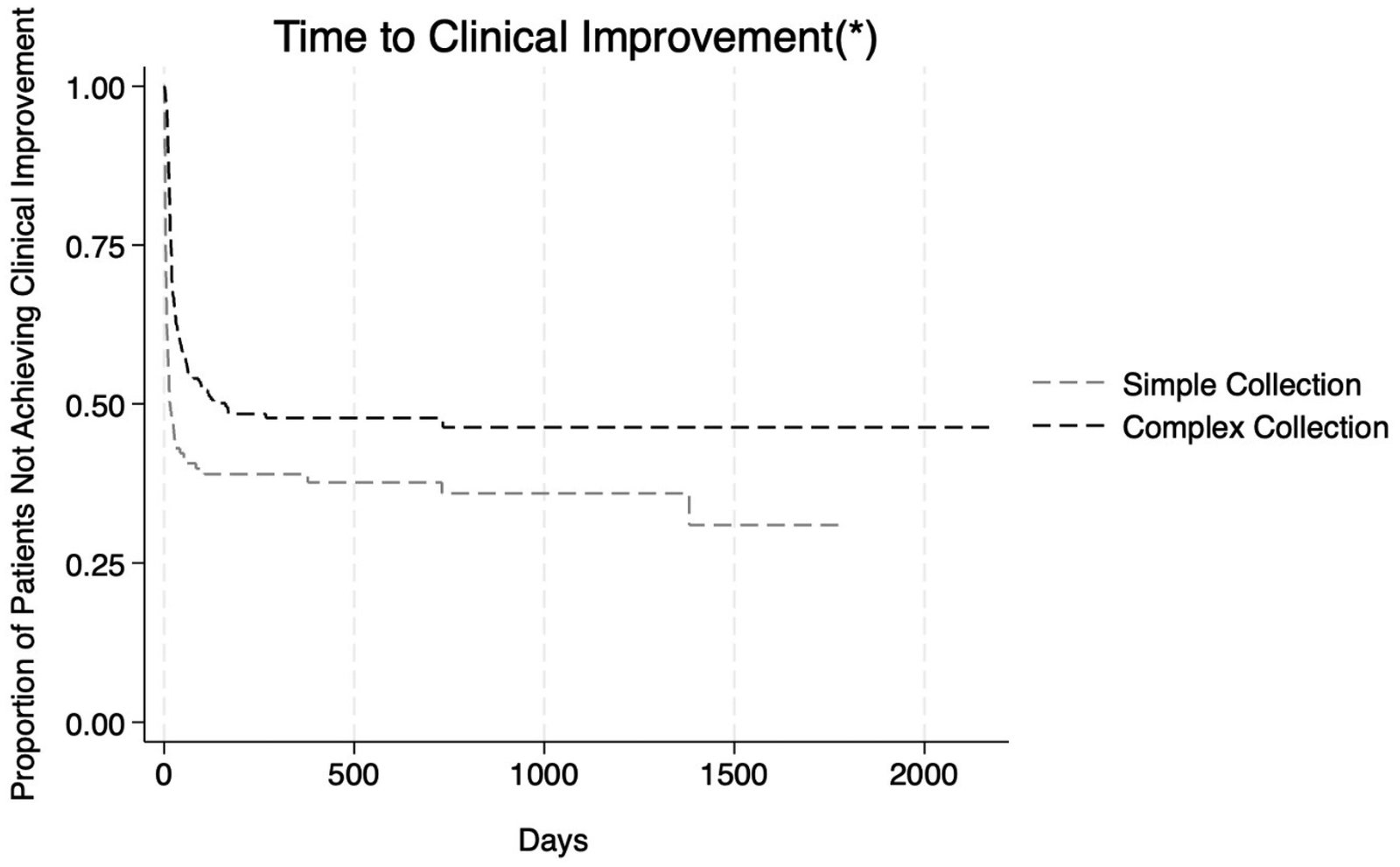

**Funding Agencies:**

None

**Table:** 

	Hazards Ratio	95% CI interval	p-value
LAMS or BFMS placement (adjusted for PS) vs. other drainage modalities	1.95	1.32-2.90	0.001
Length of Hospital Stay	0.99	0.98-0.99	<0.001
Complex collection vs. simple collection	0.30	0.21-0.44	<0.001
Etiology of pancreatitis: Alcohol-related vs. others	1.37	1.07-1.74	0.01

Significance (*) is indicated by p < 0.05; PS: propensity score to receive a LAMS/BFMS for drainage.

